# The influence of innate and adaptative immune responses on the differential clinical outcomes of leprosy

**DOI:** 10.1186/s40249-016-0229-3

**Published:** 2017-02-06

**Authors:** Adriana Barbosa de Lima Fonseca, Marise do Vale Simon, Rodrigo Anselmo Cazzaniga, Tatiana Rodrigues de Moura, Roque Pacheco de Almeida, Malcolm S. Duthie, Steven G. Reed, Amelia Ribeiro de Jesus

**Affiliations:** 1 Department of Medicine, Molecular Biology Laboratory, University Hospital, Universidade Federal de Sergipe, São Cristóvão, Sergipe Brazil; 2 Infectious Disease Research Institute, Seattle, USA; 3 Instituto de Investigação em Imunologia, Institutos Nacionais de Ciência e Tecnologia, CNPq, São Paulo, SP Brazil

**Keywords:** Leprosy, Clinical presentation, Immunology, Innate immunity, Immune pathogenesis

## Abstract

Leprosy is a chronic infectious disease caused by *Mycobacterium leprae*. According to official reports from 121 countries across five WHO regions, there were 213 899 newly diagnosed cases in 2014. Although leprosy affects the skin and peripheral nerves, it can present across a spectrum of clinical and histopathological forms that are strongly influenced by the immune response of the infected individuals. These forms comprise the extremes of tuberculoid leprosy (TT), with a *M. leprae*-specific Th1, but also a Th17, response that limits *M. leprae* multiplication, through to lepromatous leprosy (LL), with *M. leprae*-specific Th2 and T regulatory responses that do not control *M. leprae* replication but rather allow bacterial dissemination. The interpolar borderline clinical forms present with similar, but less extreme, immune biases. Acute inflammatory episodes, known as leprosy reactions, are complications that may occur before, during or after treatment, and cause further neurological damages that can cause irreversible chronic disabilities. This review discusses the innate and adaptive immune responses, and their interactions, that are known to affect pathogenesis and influence the clinical outcome of leprosy.

**Electronic supplementary material:**

The online version of this article (doi:10.1186/s40249-016-0229-3) contains supplementary material, which is available to authorized users.

## Multilingual abstracts

Please see Additional file 1 for translations of the abstract into the six official working languages of the United Nations.

## Introduction

Leprosy is a human chronic infectious disease caused by the bacillus *Mycobacterium leprae*. It is an ancient affliction that continues to have a significant global impact with official reports from 121 countries across five WHO regions recording 213 899 newly diagnosed cases in 2014 [1].

*M. leprae* is an alcohol acid-resistant bacilli with a remarkedly slow replication rate that, to date, has eluded attempts to culture it axenically in vitro [2, 3]. Although leprosy affects the skin and peripheral nerves and can cause irreversible impairment of nerve function and chronic disability, it is believed that the main route of *M. leprae* transmission is via the airways [4]. However, anecdotal reports strongly suggest that trauma-related transmission is likely, and there is also the strong suggestion of zoonotic leprosy cases resulting from contact with armadillos and the demonstration of environmental reservoirs such as water sources and amoebal cysts [5, 6].

Leprosy patients can present across an extremely wide spectrum. The Ridley and Jopling classification involves clinical, pathological, bacilloscopic, and immunological criteria, allowing a thorough characterization of six forms: the polar tuberculoid (TT) and lepromatous leprosy (LL) forms, as well the intermediate borderline tuberculoid (BT), borderline borderline (BB), and borderline lepromatous (BL) forms [7–9]. A sixth classification, indeterminate leprosy (IL), is also commonly used.

Even after treatment, patients require regular follow-up as they often present with tissue-damaging inflammatory leprosy reactions or may already have permanent neurologic deficit [10]. The spectral nature of the disease is closely associated with the type of immune response in the infected individual, making it an attractive system to investigate the immune regulation and pathogenic mechanisms, as well as the influence of host genetics upon these [10, 11]. Indeed, studies over the past 30 years have identified various determinants of leprosy and have illuminated the contribution of immunopathogenesis to disease. Many gaps remain in our knowledge and an improved understanding would provide insight toward not only leprosy but other infectious and immune-mediated diseases. This review outlines the current understanding of the innate and adaptive immune responses against *M. leprae* and their role in determining disease outcome.

## Immunopathogenic mechanisms of differing leprosy presentations

The cardinal signs of leprosy are skin lesions with altered sensation, thickened peripheral nerves, and the presence of alcohol acid-resistant bacilli. According to the World Health Organization (WHO) classification, based on smear examination or the number of lesions at diagnosis, the patients are classified into two operational groups that guide treatment: multibacillary (MB, more than five skin lesions or positive smear) and paucibacillary (PB, less than 5 lesions) [12]. Skins lesions from the extreme PB form, TT, are hypopigmented, well-bordered and with a low bacillary load. The extreme MB form, LL, is characterized by poor granuloma formation, several infiltrated skin lesions with high bacterial load. Bordeline leprosy is characterized by multiple irregular and coalescent lesions, with a ‘Swiss cheese’ aspect and usually positive baciloscopy [13]. PB leprosy patients are treated for 6 months with a cocktail consisting of rifampicin and dapsone. Due to their increased infection status, MB leprosy patients are treated for 12 months with clofazimine in additon to rifampicin and dapsone.

Polarization of the immune response specific to *M. leprae* is an important element in the pathogenesis of leprosy and in determining the clinical manifestation. A T helper (Th) 1 cytokine response has been documented at the lesional levels of TT, while a Th2 cytokine response are associated to LL forms of leprosy [14]. The immune response of TT patients is characterized by a Th1 cytokine response (interferon gamma [IFN-γ], interleukin (IL)-2, IL-15, and tumor necrosis factor [TNF]), vigorous T-cell responses to *M. leprae* antigens, and containment of the bacilli in well-formed granulomas [2, 15]. In TT lesions, macrophages are activated so that they resemble epithelial cells (at this point, they are called “epithelioid cells”), and CD4^+^ T cells are the predominant cell type. There is little evidence of *M. leprae*-specific humoral immunity [15, 16]. In contrast, the immune response of LL patients is characterized by a Th2 immune profile with production of IL-4 and IL-10 and activation of T regulatory cells (T reg), robust but not protective antibody production including formation of immune complexes, and failure to restrict *M. leprae* growth. Compared to TT, lesions from LL patients are relatively deficient in CD4+ T cells, but rather have numerous CD8^+^ T cells and macrophages heavily infected with bacilli that develop a characteristic foamy appearance [15–19]. Palermo et al. reported a higher number of Tregs and greater expression of IL-10 and cytotoxic T lymphocyte antigen-4 (CTLA-4) in LL lesions than TT lesions [20].

The balance of Th1/Th2 responses alone, however, cannot fully explain the response in leprosy. Other T cell subsets, such as T regulatory and Th17 cells, have been identified as having important roles in determining host immunity. FoxP3 positive regulatory T cells (Treg) producing TGF-β can suppress effector T cell function and were increased in stable lepromatous patients, which may explain the anergy associated with this leprosy clinical form [21]. Conversely, Th17 cells produce IL-17A, IL-17F, IL-21 and IL-22, leading to tissue inflammation and destruction, neutrophil recruitment, activating macrophages, and enhancing Th1 effector cells [21–23]. Th17 cells were first identified in experimental encephalitis and subsequently in rheumatoid arthritis, leishmaniasis and tuberculosis [21, 23]. Although several studies have demonstrated a protective role of IL-17 against other intracellular pathogens and associated diseases, relatively few reports have investigated the role of these cytokines in leprosy [21, 24]. Okada and colleagues (2015) studied families with susceptibility to *Candida albicans* and *Mycobacterium* infection and described a bi-allelic RORC mutation that resulted in the absence of IL-17A/F–producing T cells in these individuals, an impaired IFN- response to *Mycobacterium* [24]. Sadhu and colleagues demonstrated that Th17 cells are more frequent in BT and TT patients, as compared to BL and LL patients, and these cells potentiate IFN-γ production and inhibit IL-10 production by T regulatory cells. This suggests that Th17 cells also have a protective function against *M. leprae* infection [15].

The borderline forms are immunologically dynamic. There is a mixed histopathological aspect and a progressive reduction of the cell-mediated response from the BT to the BB and BL forms, accompanied by more numerous neurocutaneous lesions and increased bacterial load [4].

In an unusual presentation of leprosy, 5–15% of patients can present with a pure neuritic form (PNL) characterized by asymmetric involvement of peripheral nerves, but absence of cutaneous manifestations. This condition may be manifested as paresthesia or anesthesia, or a change in muscle strength [20].

## Contribution of early events in defining clinical outcome

**Fig. 1:**
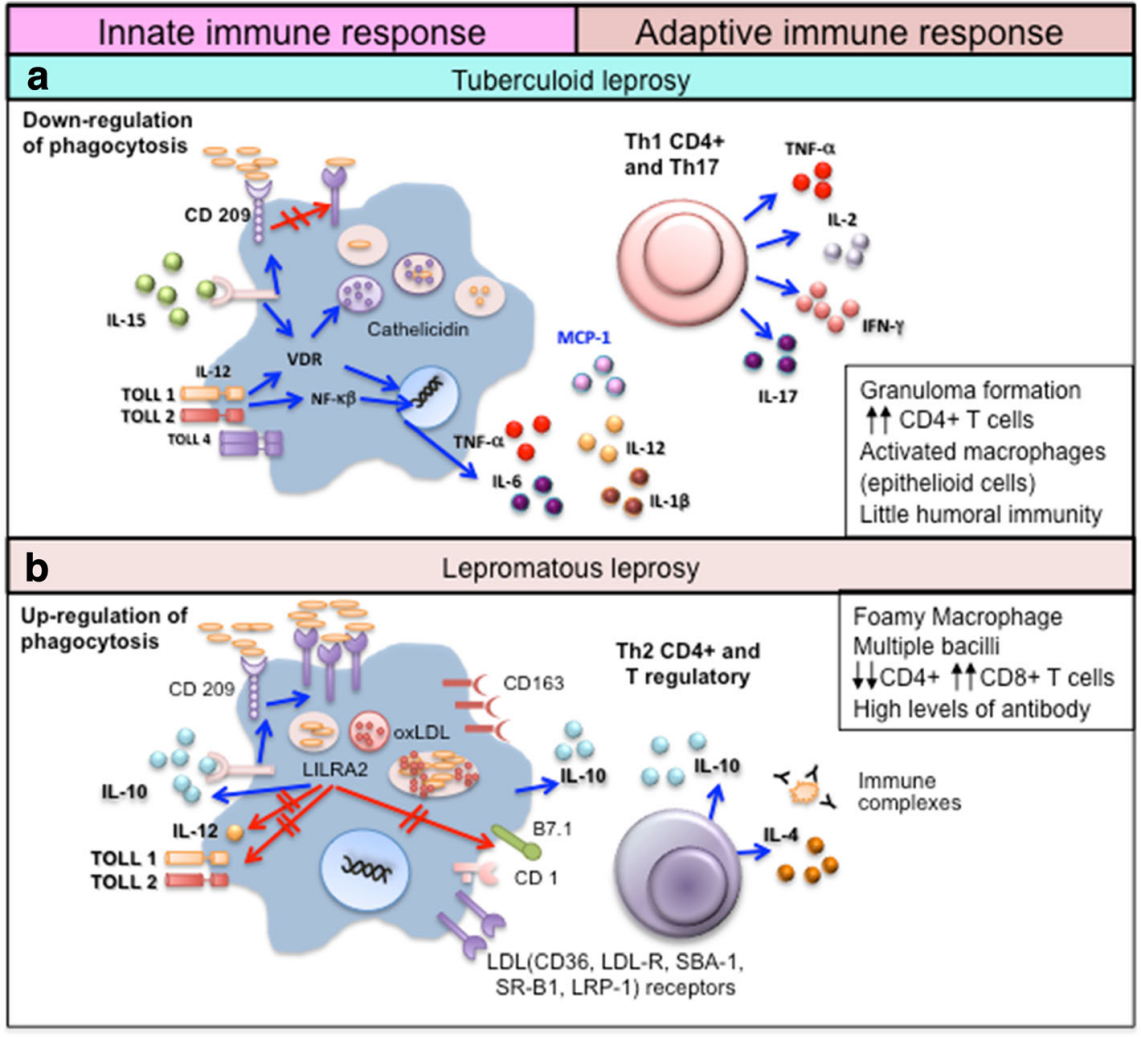
Immune response in the polar clinical forms of leprosy. **a** In tuberculoid leprosy (TT) patients, the innate immune response is activated by *M. leprae* through toll-like receptors (TLR2/1). IL-15 stimulates the vitamin D-dependent antimicrobial program in macrophages and inhibits phagocytosis of mycobacteria. These events promote a Th1 T-cell cytokine response (IFN-γ, IL-2, TNF, and IL-15) that contains the infection in well-formed granulomas, and a Th17 response (IL-17A, IL-17F, IL-21 and IL-22) that leads to tissue inflammation and destruction, neutrophil recruitment, macrophage activation, and enhancement of Th1 effector cells. **b** In lepromatous leprosy (LL) patients, IL-4, IL-10, leukocyte immunoglobulin-like receptor subfamily A member 2 (LILRA2), and oxidized phospholipids inhibit TLR2/1-induced cytokine responses but preserve IL-10 release. In addition, immune complexes trigger IL-10 production and increase phagocytosis of *M. leprae,* ApoB, haptoglobin-hemoglobin complex and oxidized phospholipids by macrophages through the receptors CD209 and CD163, without activating the vitamin D-dependent antimicrobial pathway. The foamy appearance of macrophages is due to the accumulation of lipid droplets (LD) inside these cells. There is an upregulation of perilipin and the adipose differentiation-related protein in the endoplasmic reticulum–Golgi complex with the formation of vesicles containing lipids, phospholipids, cholesterol ester, and cholesterol. Further, there is an increase in both the synthesis of LDL receptors (such as CD36, LDL-R, SBA-1, SR-B1, and LRP-1) and uptake of endogenous cholesterol that accumulates intracellularly. This induces a Th2 and Treg immune profile, with the production of IL-4 and IL-10, antibody production, absence of granulomas, and failure to restrict *M. leprae* growth [26, 31, 32, 41–46]

The innate immune response appears critical in defining the course of *M. leprae* infection and, ultimately, the clinical outcome (Fig. 1). *M. leprae* bacilli are initially recognized by several innate immune receptors, including Toll-like receptors (TLR). *M. leprae* predominantly activates the TLR2/1 heterodimer expressed in macrophages of the skin, which mediates cell activation to initiate killing of *M. leprae*. TLR2 and TLR1 are more strongly expressed in lesions of the localized TT form as compared with the disseminated LL form of the disease [25–28]. Schwann cells can also express TLR2 and the activation of TLR2 on these cells contributes to nerve damage in leprosy [29].

Cytokines such as IL-15 and IL-10 are differentially produced during the innate immune response and are known to regulate macrophage function. IL-15 is expressed in TT lesions and induces antimicrobial activity and the vitamin D-dependent antimicrobial program in macrophages, resulting in phagocytosis of mycobacteria that restricts the ability to establish infection [30]. In LL patients, IL-4 both downregulates TLR2/1 expression and inhibits the TLR2/1-induced cytokine response of macrophages. While IL-10 has no direct effect on TLR2/1 expression, it can strongly inhibit TLR2/1-induced cytokine release [26].

Activation of leukocyte immunoglobulin-like receptor subfamily A member 2 (LILRA2), expressed in several immune cells including macrophages, may control the ability of the innate immune system to activate the adaptive T cell response. Although the ligand for LILRA2 has not been identified, its activation inhibits TLR2/1-induced IL-12 release but maintains IL-10 release, and while the mechanism of LILRA2 activation during leprosy remains uncertain, LILRA2 is notably more highly expressed in LL than in TT lesions [31]. The LILRA2-expressing cells identified in LL lesions belong to a monocyte/macrophage lineage and co-express CD209, which is essential in mediating the uptake of mycobacteria by macrophages. Accordingly, expression of CD209 increases the uptake of *M. leprae*, resulting in higher bacterial loads. Similarly, oxidized phospholipids inhibit TLR2/1-induced IL-12 release, but preserve IL-10 release [32]. Immune complexes, which are abundant in the LL form due to the large quantity of antibodies that are produced, can trigger macrophages to produce IL-10 [33, 34].

The phagocytic program induced in macrophages by IL-10 is most apparent in leprosy patients that progress to the extreme LL clinical form [33, 34]. IL-10-stimulated macrophages enhance phagocytosis of both oxidized low-density lipoprotein and mycobacteria, but without triggering the vitamin D-dependent antimicrobial pathway. This divergence between the phagocytic and antimicrobial pathways likely promotes an intracellular environment that favors mycobacterial survival. IL-10-programmed macrophages are characterized by high expression of C-type lectin receptors (CD209 and CD206) and scavenger receptors (CD163, SR-A, CD36, and MARCO). CD163 mediates the uptake of hemoglobin-haptoglobin complex, thus providing a source of iron for mycobacterial survival [35], and triggers further IL-10 production [36]. All of these C-type lectin and scavenger receptors are implicated in the uptake of apoptotic cells, apoprotein B (ApoB), lipids and lipoproteins, that are nutrient sources for *M. leprae* [37], and are also associated with functions related to the maintenance of tissue homeostasis by macrophages [37–40]. Lipid uptake also inhibits the innate immune response against the bacteria by diminishing TLR-induced antimicrobial activity and by skewing the cytokine balance toward IL-10 secretion whilst inhibiting IL-12 production [41]. This IL-10-derived macrophage pathway is found in the MB forms and enhances phagocytosis of oxidized phospholipids and additional *M. leprae*. Studies have shown a colocalization of the CD209 and CD163 markers, *M. leprae*, apoprotein B, and host-derived oxidized phospholipids within the phagosomes [26, 41]. Biopsies from LL patients exhibit macrophages that are packed with lipid droplets (LD), named “foamy macrophages” [42, 43]. Additional mechanisms such as enhanced cell survival through decreased apoptosis may also contribute to the foamy macrophage characteristic of LL lesions [44]. Schwann cells (SC) from LL patients also have a foamy phenotype, and LD accumulation seems to be associated with the pathophysiology of leprosy [45, 46].

As the most efficient antigen-presenting cells, dendritic cells (DC) play an important role in connecting innate and adaptive immunity but the actual contribution of DC subsets to the pathogenesis of leprosy remains controversial. Whereas some studies have reported a larger number of DC in the lesions of TT patients [47, 48], others have suggested that plasmocytoid DC are not involved in host responses against *M. leprae* [49, 50].

## Immunopathogenesis of leprosy reactions

In the complex evolution of leprosy, two types of spontaneous acute inflammatory phenomena, are known to occur. These “leprosy reactions” occur in 30–50% of patients at some time during the course of their disease [51, 52]. Reactions can present with intense neural inflammation, resulting in sudden and even permanent loss of sensory, autonomic and motor functions. Besides aggravating the neural lesions, reactions frequently require prolonged treatment with toxic drugs such as corticosteroids and/or thalidomide, which is a major concern for the leprosy patients.

**Fig. 2:**
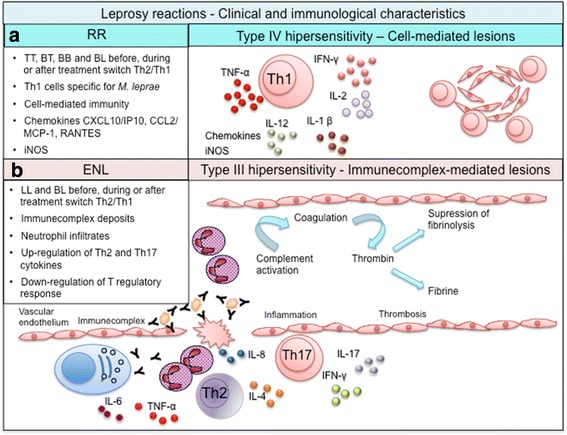
Immunological aspects of leprosy reactions. **a** RR represents a type IV hypersensitivity reaction. Sudden activation of an inflammatory response to *M. leprae* antigens, often after the initiation of treatment, triggers a transient conversion from a Th2 toward a Th1 response. The cytokine expression pattern in lesions indicates enhancement of the Th1 response along with activation of the innate response and inflammatory products. **b** ENL involves high levels of TNF, immune complex-associated vasculitis, and intralesional infiltration of neutrophils, eosinophils, and CD4+ T cells. ENL is initiated by the deposition of immune complexes and activation of complement, triggering elevation of several pro-inflammatory cytokines, neutrophilic infiltrates, and vasculitis [61–64, 82, 83]

Reactions are classified into two main types: Type I reactions, also commonly known as reversal reaction (RR), and Type II reaction, commonly known as erythema nodosum leprosum (ENL) [51, 52]. The mediators of tissue damage in these reactions are partially known, with increased amounts of Th1 cytokines such as IFN-γ, IL-12, and IL-2 clearly demonstrated in both RR and ENL [51, 53] (Fig. 2). It remains unclear, however, whether the inflammatory profile observed in the lesions or blood during reactions is the cause or consequence of these reactions.

## Type I reactions (RR)

RR occurs in 30% of patients and involve a sudden activation of an inflammatory response to *M. leprae* antigens. RR are the main cause of nerve damage in leprosy and occur most frequently after the initiation of treatment, most often arising in the first two months after the initiation of chemotherapy. This reflects a switch from a Th2-predominant toward a Th1 response [17, 53–57]. Affected patients present with swollen hands and feet, exacerbation of cutaneous lesions and neural involvement, that can result in hospitalization [55, 58].

Both innate and adaptive immune responses participate in the pathogenesis of RR. RR lesions are associated with a type-IV (or delayed-type) hypersensitivity reaction and immunophenotyping studies have indicated that the number and percentage of CD4+ T cells are increased in reacting skin lesions [4, 59, 60]. The vitamin D-dependent antimicrobial pathway is activated, and IL-1, IL-2, IL-6, IL-8, IL-12 p40, IFN-γ, TNF, IL-2 receptor [61], and CXC chemokine-10 (CXCL10 or IP10) are detected in the circulation and cutaneous lesions [60]. The tumor necrosis factor superfamily (TNFSF) is essential for the induction of programmed cell death and costimulation of distinct cell types [62, 63] and the TNFSF15 locus has been associated with susceptibility for leprosy in Chinese individuals. A recent study from Brazil and Vietnam reported that the TNSF8 locus, but not the TNFSF15 locus, confers susceptibility to RR [62]. Overall, the cytokine expression pattern in the RR lesions indicates enhancement of the Th1 response with accompanying, or related, activation of the innate immune response and inflammatory products.

## Type II reactions (ENL)

ENL affects patients with poor cellular immune responses but who have preserved humoral responses, and thus presents in MB patients with high levels of anti-*M. leprae* immunoglobulins. ENL is characterized by an abrupt onset of erythematous and painful nodules accompanied by systemic symptoms such as fever, lymph node infarction, bone tenderness and hepatosplenomegaly. Neuritis can persist for years as a chronic and recurrent symptom in most patients [64].

ENL is usually initiated by deposition of immune complexes and activation of the complement cascade, resulting in vasculitis or a type-III hypersensitivity reaction [7]. High immunoglobulin levels and low levels of complement components (a sign of complement activation) can be detected along with the presence of platelet-derived growth factor BB (PDFG-BB) [60, 65]. PDFG-BB is known to promote angiogenesis and is a potential marker of ENL [65].

ENL lesions present with deposits of immunoglobulins, complement and some mycobacterial constituents [66], along with expression of IL-6, IL-8, and IL-10 mRNA and sustained expression of IL-4 and IL-5 mRNA, consistent with neutrophil chemotaxis and antibody production [67]. Tissue infiltration by CD4 cells and neutrophils occurs [64]. The same cytokines as mentioned earlier for RR are also found at high levels in the plasma during ENL, but in ENL there are significantly higher levels of IL-4, IL-5, IL-10, IL-6, IL-7 and TNF [60, 61, 68–72]. The most severe reactions are associated with increased production of TNF and IFN-γ and IFN-γ injections have been shown to activate ENL lesions [52, 73, 74]. While FoxP3 expressing Treg producing TGF-β are increased in stable lepromatous leprosy patients, patients with reactions exhibit an imbalance in Th17 and Treg populations [22]. These data suggest that Treg may exert control on the inflammatory response during leprosy reactions.

Moreover, Vieira et al. determined either the frequency of circulating Tregs in patients with RR and ENL or the frequency of Tregs and interleukin IL-17, IL-6, and (TGF)-β-expressing cells not only peripheral blood but in biopsies taken before and during the reaction episodes. Their results suggest that in ENL, downmodulation of Tregs may influence the development of Th-17 responses that characterize this reaction [75].

Early diagnosis of leprosy reactions is crucial for efforts to reduce tissue damage and prevent disabilities. Khadge and colleagues (2015) showed that in newly diagnosed patients from Bangladesh, Brazil, Ethiopia and Nepal the production IFN-γ, IP-10, IL-17 and VEGF in supernatants from *M. leprae* antigen-stimulated cells increased during type 1 reaction, as compared to patients without leprosy reactions [76]. There is, however, a lack of biomarkers that are capable of reliably predicting reactions within endemic populations [76].

## Neuropathy

Nerve injury is the hallmark of progressive *M. leprae* infection and is present in all forms of leprosy [77–79]. Physical impairment in leprosy is defined as any reduction in sensory or motor functions. Since neurological involvement is inherent to all forms of leprosy, disability is a frequent complication, resulting from the natural course of disease [58]. The major determinant of neuronal injury is the ability of *M. leprae* to bind and infect SC. *M. leprae* phenolic glycolipid (PGL)-I interacts with the laminin-2 receptor located on the SC membrane [53, 80–82] and laminin-binding protein 21 (LBP21) mediates the intracellular entry of *M. leprae* into the SC [82, 83].

In TT patients, neural damage has a direct positive correlation with IFN-γ [67]. The activated Th1 response and development of strong cellular immunity contribute to the formation of tuberculoid granulomas and caseous necrosis, and may culminate in the appearance of abscesses and complete destruction of the nerves [4]. In contrast, in LL patients exhibiting Th2 responses, the neuropathy is directly related to the *M. leprae* infection of peripheral nerves.

## Conclusions

Our current knowledge postulates that the initial interaction between the *M. leprae* and the host innate immune response impacts the initial growth and establishment of infection, then potentially influencing the type of adaptive immune response that is induced against the infection. Although considerable progress has been made in understanding leprosy and the factors involved in its clinical outcomes, an improved understanding of the early events of *M. leprae* infection are needed. This will hopefully help us better understand the diverse pathogenic events that can occur later in infection, to predict clinical outcomes and risk for complications, make improvements in drug design and individualized therapies possible, and to reveal the potential for novel immunotherapies. Given its spectral presentation leprosy is an instructive human disease that allows for direct and controlled comparison of immune responses, in particular, CD4 T cell differentiation and discoveries in leprosy can therefore provide critical insight that can be applied to other immune- and pathogen-mediated diseases.

## Additional file

Additional file 1: Multilingual abstracts in the six official working languages of the United Nations. (PDF 1008 kb)

## Abbreviations

ApoB: Apoprotein B
BB: Borderline borderline
BL: Borderline lepromatous
BT: Borderline tuberculoid
CTLA-4: Cytotoxic T lymphocyte antigen-4
ENL: Erythema nodosum leprosum
IFN-γ: Interferon gamma
IL: Indeterminate leprosy
IL: Interleukin
IL-10: Interleukin-10
LBP21: Laminin-binding protein 21
LD: Lipid droplets
LILRA2: Leukocyte immunoglobulin-like receptor subfamily A member 2
LL: Lepromatous leprosy
MB: Multibacillary
PB: Paucibacillary
PGL: Phenolic glycolipid
PNL: Pure neuritic form
RR: Reversal reaction
SC: Schawnn cell
T regs: T regulatory cells
Th1 cell: T helper 1cell
Th17: T helper 17 cell
Th2 cell: T helper 2 cell
TLRs: Toll-like receptors
TNF: Tumor necrosis factor
TT: Tuberculoid leprosy
WHO: World Health Organization
