# Supplementary material for: The influence of innate and adaptative immune responses on the differential clinical outcomes of leprosy
Source: Infect Dis Poverty. 2017 Feb 6;6:5. doi: 10.1186/s40249-016-0229-3 (PMC5292790; doi:10.1186/s40249-016-0229-3)
Supplement: Additional file 1: — Multilingual abstracts in the six official working languages of the United Nations. (PDF 1008 kb) [file 40249_2016_229_MOESM1_ESM.pdf]

Translation of the abstract into the six official working languages of the United Nations

أثر الاستجابات المناعية المكتسبة و الموروثة في النتائج الإكلينيكية المختلفة لمرض الجذام.

أدريانا باربوسا دي ليما فونسيكا و ماريز دوفال سيمون و رودريجو أنسيلموكازانيجا و تاتانيا رودريجوز دي مورا وروكو باتشيكو دي الميدا و مالكوم أس دوثي وستيفين جي ريد وإيمليا ريبيرو دي جيسوس

تلخيص

الجذام مرض معدي مزمن تسببه بكتيريا تسمى *مايكوباكتيريوم ليبرا* . وطبقا للتقارير الرسمية من 121 دولة عبر مناطق منظمة الصحة العالمية الخمس، تم تشخيص 213,899 حالة إصابة جديدة خلال عام 2014. على الرغم من أن الجذام يصيب الجلد والأعصاب الطرفية إلا إنه يمكن أن يوجد في نطاق من الأشكال الإكلينيكية و الهيستوباثولوجية والتي تتأثر بشدة بالاستجابة المناعية للمصاب. وتشبه تلك الأشكال الحالات المتطرفة من جذام التيوبركلويد ليبروسي (TT) مع *إم ليبار* *M. leprae* وتحديدا Th1 ولكن أيضا Th17. الاستجابة التي تحد من تضاعف بكتيريا *M. leprae* خلال الليبروماتيوز ليبروسي (LL) مع *M. leprae* - تحديدا Th2 و الاستجابات المعتادة لـT والتي لا تتحكم في إعادة إنتاج *M. leprae* ولكن على الأحرى تسمح بالتحلل البكتيري. وتقدم الخطوط الحدودية التقاطعية التي تقدمها الأشكال الإكلينيكية مع استجابات مناعية متشابهة ولكن أقل حدة تحيزات مناعية. دورات الالتهاب الحاد المعروفة بردود فعل الجذام هي تعقيدات قد تحدث قبل أو أثناء أو بعد العلاج وتسبب مزيد من التلف العصبي والذي يمكن أن يصيب بإعاقات مزمنة غير قابلة للشفاء. تناقش هذه المراجعة الاستجابات المناعية الفطرية والمكتسبة و تداخلاتها المعروفة في تأثيرها على الداء وتأثيرها على النتائج الإكلينيكية لمرض الجذام.

Translated from English version into Arabic by Mohamed Gaafar, through

先天性和适应性免疫反应对不同的麻风病临床结果的影响

Adriana Barbosa de Lima Fonseca, Marise do Vale Simon, Rodrigo Anselmo Cazzaniga, Tatiana Rodrigues de Moura, Roque Pacheco de Almeida, Malcolm S Duthie, Steven G Reed, Amelia Ribeiro de Jesus

摘要

麻风病是由麻风分枝杆菌(Mycobacterium leprae)引起的慢性传染病。世界卫生组织 5 个区域 121 个国家的官方报告称，2014 年有 213 899 例新诊断病例。虽然麻风病影响皮肤和末稍神经，它也可以呈现为一系列的临床和组织病理学表现，严重影响感染个体的免疫应答。这些表现包括结核样型麻风（TT）极值，包括麻风分支杆菌特异性 Th1 和 Th17，其反应限制麻风分支杆菌的增殖，直到发展为瘤型麻风（LL）。LL 的麻风分支杆菌特异性 Th2 和调节性 T 细胞免疫应答不控制麻风分支杆菌繁殖，而允许细菌传播。不同极型间的临床表现形式相似，但有不太极端的免疫偏倚。急性炎症发作即麻风反应，是麻风病的并发症，可能发生在治疗之前、期间或之后，并可进一步引发神经损伤，造成不可逆的慢性残疾。本综述讨论了先天性和适应性免疫反应及其相互作用，即影响发病机制和麻风的临床预后。

Translated from English version into Chinese by Jin Chen, edited by Pin Yang

L'influence des réponses immunitaires adaptatives et acquises sur les résultats cliniques différentiels de la lèpre

Adriana Barbosa de Lima Fonseca, Marise do Vale Simon, Rodrigo Anselmo Cazzaniga, Tatiana Rodrigues de Moura, Roque Pacheco de Almeida, Malcolm S Duthie, Steven G Reed, Amelia Ribeiro de Jesus

Résumé
La lèpre est une maladie infectieuse chronique due à *Mycobacterium leprae (M. leprae).* Selon des rapports officiels publiés par 121 pays répartis dans cinq régions de l'OMS, il y a eu 213 899 nouveaux cas diagnostiqués en 2014. Cette maladie touche la peau et les nerfs périphériques, mais elle peut se manifester sous diverses formes cliniques et histopathologiques qui sont fortement influencées par la réponse immunitaire de la personne infectée. Ce polymorphisme se distingue en deux pôles, la lèpre tuberculoïde (TT), caractérisée par une réponse des cellules Th1 spécifiques à *M. leprae*, mais aussi des cellules Th17, qui limitent la multiplication de *M. leprae*, et la lèpre lépromateuse (LL), caractérisée par une réponse des cellules Th2 spécifiques à *M. leprae* ainsi qu'une réponse de régulation des cellules T qui ne permettent pas de contrôler la réplication de *M. leprae*, mais entrainent plutôt la prolifération de la bactérie. Les formes intermédiaires cliniques dites *borderline* interpolaires présentent des polarisations immunitaires semblables, mais moins extrêmes. Des manifestations inflammatoires aiguës, ou réactions lépreuses, sont des complications qui peuvent se produire avant, pendant ou après le traitement et causer d'autres dommages neurologiques qui peuvent entrainer des incapacités chroniques irréversibles. Cette étude porte sur les réponses immunitaires adaptatives et acquises, ainsi que sur leurs interactions, connues pour leurs impacts sur la pathogenèse et les résultats cliniques de la lèpre.

Translated from English version into French by Sabiha Azibi, through

TRANSLATORS
WITHOUT BORDERS

Воздействие врожденных и адаптационных иммунных ответов на дифференциальный клинический исход лепры

Адриана Барбоса-де-Лима-Фонсека, Марис До-Вале-Симон, Родриго Ансельмо-Каззанига, Татьяна Родригез-де-Маура, Роке Рачеко-де-Альмейда, Малькольм С. Дати, Стивен Дж. Рид, Амелия Рибейро-де-Хесус

Краткое изложение
Лепра – это хроническое инфекционное заболевание, вызываемое микобактериями Mycobacterium leprae. По данным официальных исследований в 121 стране по пяти регионам, охватываемым Всемирной организацией здравоохранения, в 2014 году было поставлено 213 899 новых диагнозов лепры. Хотя лепра поражает кожу и периферические нервы, она может проявляться в самых различных клинических и гистопатологических формах, которые сильно связаны с иммунной реакцией пациента. Эти формы включают в себя такие экстремальные случаи, как туберкулоидная лепра (TT), с M. leprae-специфическим Th1, а также Th17, иммунным ответом, ограничивающим размножение M. leprae, а также лепроматозную лепру (LL), с M. leprae-специфическими Th2 и T ответами, не контролирующими репликацию M. leprae,, а позволяющими распространение бактерий. Биполярные пограничные клинические формы демонстрируют похожие, но менее экстремальные иммунные ошибки. Периоды острого воспаления, называемые лепрозными реакциями, являются осложнениями, которые могут происходить до лечения, во время или после него, и приводят к дальнейшим неврологическим повреждениям, которые могут вызвать необратимую хроническую инвалидность. Данный обзор рассматривает врожденные и адаптационные иммунные ответы и их взаимодействие, влияющие на патогенез и клинический исход заболевания.

Translated from English version into Russian by Elena McDonnell, through

TRANSLATORS
WITHOUT BORDERS

La influencia de las respuestas inmunitarias innata y adaptativa sobre los diferentes desenlaces clínicos de la lepra

Adriana Barbosa de Lima Fonseca, Marise do Vale Simon, Rodrigo Anselmo Cazzaniga, Tatiana Rodrigues de Moura, Roque Pacheco de Almeida, Malcolm S Duthie, Steven G Reed, Amelia Ribeiro de Jesus

Resumen

La lepra es una enfermedad infecciosa crónica causada por el *Mycobacterium leprae*. Según informes oficiales de 121 países pertenecientes a cinco regiones de la OMS, se produjeron 213.899 nuevos casos diagnosticados en 2014. Aunque la lepra afecta a la piel y a los nervios periféricos, puede presentarse a lo largo de un espectro de formas clínicas e histopatológicas altamente influenciadas por la respuesta inmunitaria del individuo afectado. Estas formas incluyen los extremos de la lepra tuberculoide (TT), con una respuesta Th1 específica para *M. leprae*, pero también con una respuesta Th17, que limita la multiplicación de *M. leprae*, hasta la lepra lepromatosa (LL), con una respuesta Th2 específica de *M. leprae* y una respuesta T reguladora, que no controlan la replicación de *M. leprae* sino que permiten la diseminación bacteriana. Las formas clínicas interpolares limítrofes presentan sesgos inmunológicos similares, aunque menos extremos. Los episodios inflamatorios agudos, conocidos como reacciones leprosas, son complicaciones que pueden tener lugar antes de, durante o después del tratamiento y que pueden causar daños neurológicos que pueden dar lugar a discapacidades crónicas irreversibles. Esta revisión analiza las respuestas inmunitarias innata y adaptativa y las interacciones entre ellas, de las.

Translated from English version into Spanish by Alicia Pallas, through

TRANSLATORS
WITHOUT BORDERS
